# Structural and Functional Studies of a Bothropic Myotoxin Complexed to Rosmarinic Acid: New Insights into Lys49-PLA_2_ Inhibition

**DOI:** 10.1371/journal.pone.0028521

**Published:** 2011-12-21

**Authors:** Juliana I. dos Santos, Fábio F. Cardoso, Andreimar M. Soares, Maeli dal Pai Silva, Márcia Gallacci, Marcos R. M. Fontes

**Affiliations:** 1 Departamento de Física e Biofísica, Instituto de Biociências, University Estadual Paulista, Botucatu/Sao Paulo, Brazil; 2 Instituto Nacional de Ciência e Tecnologia em Toxinas, Conselho Nacional de Desenvolvimento Científico e Tecnológico, Botucatu/Sao Paulo, Brazil; 3 Departamento de Farmacologia, Instituto de Biociências, University Estadual Paulista, Botucatu/Sao Paulo, Brazil; 4 Fundação Oswaldo Cruz - Rondônia and Centro de Estudos de Biomoléculas Aplicadas, Universidade Federal de Rondônia, Porto Velho/Rondonia, Brazil; 5 Departamento de Morfologia, Instituto de Biociências, University Estadual Paulista, Botucatu/Sao Paulo, Brazil; MRC National Institute for Medical Research, United Kingdom

## Abstract

Snakebite envenoming is an important public health problem in many tropical and subtropical countries, and is considered a neglected tropical disease by the World Health Organization. Most severe cases are inflicted by species of the families Elapidae and Viperidae, and lead to a number of systemic and local effects in the victim. One of the main problems regarding viperidic accidents is prominent local tissue damage whose pathogenesis is complex and involves the combined actions of a variety of venom components. Phospholipases A_2_ (PLA_2_s) are the most abundant muscle-damaging components of these venoms. Herein, we report functional and structural studies of PrTX-I, a Lys49-PLA_2_ from *Bothops pirajai* snake venom, and the influence of rosmarinic acid (RA) upon this toxin's activities. RA is a known active component of some plant extracts and has been reported as presenting anti-myotoxic properties related to bothopic envenomation. The myotoxic activity of Lys49-PLA_2_s is well established in the literature and although no *in vivo* neurotoxicity has been observed among these toxins, *in vitro* neuromuscular blockade has been reported for some of these proteins. Our *in vitro* studies show that RA drastically reduces both the muscle damage and the neuromuscular blockade exerted by PrTX-I on mice neuromuscular preparations (by ∼80% and ∼90%, respectively). These results support the hypothesis that the two effects are closely related and lead us to suggest that they are consequences of the muscle membrane-destabilizing activity of the Lys49-PLA_2_. Although the C-terminal region of these proteins has been reported to comprise the myotoxic site, we demonstrate by X-ray crystallographic studies that RA interacts with PrTX-I in a different region. Consequently, a new mode of Lys49-PLA_2_ inhibition is proposed. Comparison of our results with others in the literature suggests possible new ways to inhibit bothropic snake venom myotoxins and improve serum therapy.

## Introduction

Envenoming resulting from snakebites is an important public health problem in many tropical and subtropical countries [1], [2]. Although data on this topic are scarce, a recent study estimates that at least 421,000 envenomations and 20,000 deaths due to snakebites occur each year [2]. This problem is particularly important in the rural tropics because the populations of these areas usually have poor access to health systems and, in some cases, antivenom is scarce [1], [3]. A large number of victims survive with permanent physical and also psychological sequelae. Young agricultural workers, especially males, are the most affected group, making snakebite envenoming a truly occupational disease [1] now considered a neglected tropical disease by the World Health Organization (WHO; http://www.who.int/neglected_diseases/diseases/snakebites/en/index.html). Even though the majority of deaths due to snakebite envenoming occur in south and south-east Asia and sub-Saharan Africa [2], these accidents are also an important health problem in Latin America [4] where snakebites from the *Bothrops* genus (Viperidae family) are responsible for more than 85% of all reported ophidian accidents [5], [6]. One of the main problems associated with these events is prominent local tissue damage characterized by swelling, blistering, hemorrhaging and necrosis of the skeletal muscle which develops rapidly after the snakebite [1]. Consequently, a delay in access to health facilities frequently results in drastic tissue damage and permanent disability [1], [7]–[11].

The pathogenesis of myonecrosis is complex and involves the combined actions of a variety of venom components, such as myotoxins and metalloproteinases [12]–[18]. Nowadays, parenteral administration of animal-derived antivenoms that consist of whole IgG molecules (∼150 kDa), F(ab′)_2_ or Fab fragments [19]–[22] constitutes the only specific treatment for snakebite envenoming. But despite the success of this therapy in neutralizing toxins responsible for the systemic effects of snakebite envenomation, this antivenom presents a limited effectiveness in protecting against establishment of myonecrosis [23]. Therefore, advances in the treatment of this local pathology may be achieved by elucidating the snake venom components involved in its genesis and the molecular basis of their mechanism of action.

Phospholipases A_2_ (PLA_2_s) are the most abundant proteins found in Viperidae snake venoms [24] and, in addition to their well-established ability to hydrolyze lysophospholipids in a calcium-dependent mechanism [25], these proteins can display toxic effects by different mechanisms [26]. A recent phylogenetic study shows that snake venom PLA_2_s can be classified into two groups according to their evolutionary derivation: i) the calcium-dependent catalytically active enzymes, such as Asp49-, Asn49- and Gln49-PLA_2_s; and ii) the catalytically inactive PLA_2_s that exert their effects through a still unresolved calcium-independent mechanism (Lys49-, Arg49- and some Asp49-PLA_2_s) [27]. The former group usually includes acidic PLA_2_s that act as monomeric toxins whereas the latter includes basic PLA_2_s that adopt a homodimeric configuration [27].

Despite their lack of enzymatic activity, Lys49-PLA_2_ myotoxins play a key role in myonecrosis, given that when they are selectively neutralized, most of the muscle-damaging effect of whole venoms is prevented [28]–[32]. In addition, several other biological activities have been described for these toxins both *in vivo* and *in vitro* [15]. Although myotoxic Lys49-PLA_2_s are devoid of significant neurotoxicity *in vivo* [15], some of them are able to induce an inhibitory neuromuscular activity *in vitro* [33]–[38]. Recently, a review of experimental evidence on this topic has suggested that both the *in vitro* neuromuscular blockade and the muscle damage induced by Lys49-PLA_2_s are consequences of their general muscle membrane-destabilizing activity [38].

Studies involving the interaction of myotoxic Lys49-PLA_2_s with potential neutralizing molecules have been one of the main methodologies employed to elucidate the action mechanism and structural determinants of the biological activities of these toxins [35], [39]–[42]. Furthermore, PLA_2_ inhibitors may provide therapeutic molecular models with antiophidian properties and may be applicable as a supplement to the conventional serum therapy [42]. Medicinal plants constitute an important source of bioactive compounds able to antagonize the activity of various crude venoms and purified toxins [40], [42]–[53]. In 2005, Ticli and colleagues showed that rosmarinic acid (RA) isolated from *Cordia verbenacea* significantly inhibits the myotoxic effect induced by the two main basic PLA_2_s homologues (BthTX-I and BthTX-II) from *Bothrops jararacussu* snake venom [40]. The authors also demonstrated that RA enhances the effect of commercial equine polyvalent antivenom against isolated myotoxins or the crude venom [40].

In this work we report structural and functional studies concerning the mechanism through which RA neutralizes the *in vitro* inhibitory neuromuscular effect and muscle damage caused by PrTX-I, a Lys49-PLA_2_ from *Bothops pirajai* snake venom [54]. The data obtained show that RA is able to drastically reduce both the muscle damage and the neuromuscular blockade caused by the toxin, supporting the hypothesis that these two effects are mainly consequences of the muscle membrane-destabilizing activity of Lys49-PLA_2_s. X-ray crystallographic studies demonstrate that RA interacts with PrTX-I at the entrance of its hydrophobic channel and not at its C-terminus, a region indicated as responsible for the myotoxic effects of Lys49-PLA_2_s [15], [55]–[59]. Comparison of our results with others available in the literature lead us to suggest possible new approaches to inhibit bothropic snake venom myotoxins so as to achieve improvement in serum therapy.

## Methods

### Protein purification and Inhibitor Source

PrTX-I was isolated from *Bothrops pirajai* snake venom as previously described [54] and rosmarinic acid (RA) was purchased from Sigma-Aldrich.

### Functional Studies

#### Animals

Adult male mice weighing 25–30g were maintained under a 12 h light-dark cycle (lights on at 07:00 AM) in a temperature-controlled environment (22±2°C) for at least 10 days prior to the experiments. Food and water were freely available. Animal procedures were in accordance with the guidelines prepared by the Committee on Care and Use of Laboratory Animal Resources, National Research Council, USA.

#### Neuromuscular-blocking activity

Mice were killed by exsanguination after ether anesthesia. Phrenic-diaphragm preparation was removed and mounted vertically in a conventional isolated organ-bath chamber containing 15 mL of physiological solution of the following composition (mmol/L): NaCl, 135; KCl, 5; MgCl_2_, 1; CaCl_2_, 2; NaHCO_3_, 15; Na_2_HPO_4_, 1; glucose, 11. This solution was bubbled with carbogen (95% O_2_ and 5% CO_2_). The preparation was attached to an isometric force transducer (Grass, FT03) to record the twitch tension. The transducer signal output was amplified and recorded on a computer via a transducer signal conditioner (Gould, 13-6615-50) with an AcquireLab Data Acquisition System (Gould). The resting tension was 5 g. Indirect contractions were evoked by supramaximal pulses (0.2 Hz, 0.5 ms) delivered from an electronic stimulator (Grass-S88K) and applied to the phrenic nerve by means of a suction electrode.

The preparation was stabilized for 45 minutes before the addition of a single concentration of toxin. For inhibition experiments, a fixed amount of PrTX-I dissolved in physiological saline solution (PSS; 0.9% NaCl) was mixed with the same quantity of RA, in order to obtain a 1∶1 (w/w) toxin/RA ratio. Mixtures were incubated for 30 minutes at 35±2°C. Control experiments were performed in the absence of toxin or in the presence of RA alone. The degree of protection offered by RA after 90 minutes of contact with the preparation was expressed as a percentage of neuromuscular blockade observed in the presence of the mixture of toxin plus RA relative to the blockade seen in the presence of toxin alone.

#### Muscle-damaging activity

At the end of the functional study, phrenic-diaphragm muscle was removed from the bath and immersed in Bouin's fixative, processed, and embedded in Historesin (Kit Historesin Leica). Histological transverse sections (5 µm thick) were cut out in a microtome and stained with hematoxylin and eosin (HE) prior to examination by light microscopy [60]. Muscle samples were also fixed in Karnovsky's fixative for 4 h and washed in 1% osmium tetroxide. The tissue was dehydrated in ascending concentrations of acetone and embedded in Epon resin. Sections (1.5 µm) were stained with uranyl acetate and lead citrate, and examined by electron microscopy.

Morphological damage was quantified in HE-stained preparations, using an Imaging Analysis System (Leica, Qwin). The number of fibers with lesions was expressed as a percentage of the total number of cells (muscle damage index), in three non-overlapping non-adjacent areas of each muscle, observed at the same magnification. The degree of neutralization offered by RA was expressed as a percentage of the myonecrosis index in the presence of toxin plus RA relative to that index in the presence of the toxin alone.

#### Statistical analysis

Results are expressed as mean ± S.E. Data were analyzed by ANOVA complemented by the Tukey-Kramer test. Values of *P*<0.05 were considered significant.

### Structural Studies

#### Crystallographic studies

Co-crystallization experiments were performed with PrTX-I at a concentration of 12 mg/mL [61]. Crystals of the complex were obtained by the sitting drop method [62] after thirty minutes of protein/ligand pre-incubation. The best crystals were produced by combining 1 µl of protein solution, 0.5 µl of RA solution (35 mM) and 1 µl of reservoir solution and equilibrated against 0.5 ml of the same precipitant solution - 20% PEG4000, sodium citrate pH 5.6 and 20% 2-propanol [61].

#### X-ray data collection and processing

X-ray diffraction data were collected at a wavelength of 1.427 Å (at 100 K) using a synchrotron radiation source (MX2 station - Laboratório Nacional de Luz Síncrotron, LNLS, Campinas, Brazil) and a MAR CCD imaging-plate detector (MAR Research). A crystal was mounted in a nylon loop and flash-cooled in a stream of nitrogen at 100 K using no cryoprotectant [61]. Data were processed using the HKL program package [63].

#### Structure determination and refinement

The crystal structures were solved by molecular replacement using the program Phaser [64] and the coordinates of PrTX-I complexed with á-tocopherol [65] as a model. The model was improved, as judged by the free R-factor [66], through rounds of crystallographic refinement using the REFMAC program [67]. Manual rebuilding was performed with the COOT program [68]. The quality of the model was checked by the program Procheck [69].

#### Ligand design

The program Avogadro v.1.0.0 [70] (http://avogadro.openmolecules.net/) was used to design the RA molecule (3-(3,4-dihydroxyphenyl)-2-[(E)-3-(3, 4-dihydroxyphenyl)prop-2-enoyl]oxypropanoic acid) and improve its overall structure by an energy minimization process based on the MMFF94 force field [71]. Geometric optimization was performed with a steepest-descent algorithm (500 steps with a 10^−7^ convergence criterion).

## Results

### Neuromuscular blocking activity

**Figure 1:**
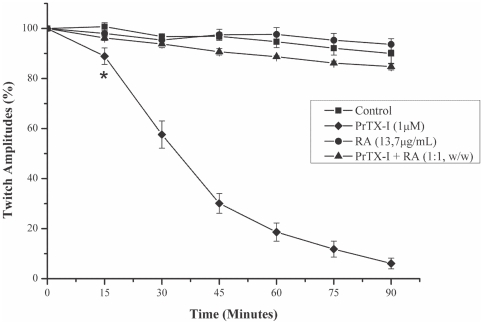
Effects of PrTX-I alone and PrTX-I pre-incubated with RA on indirectly evoked twitches in mouse phrenic-diaphragm preparations. The ordinate represents the percentage amplitude of twitches relative to the initial amplitude. The abscissa indicates the time after the addition of toxin to the organ bath. The points are the mean ± S.E. * indicates the point at which differences between PrTX-I and the control become significant.

PrTX-I (1 µM) induced a time-dependent blockade of indirectly evoked twitches in phrenic-diaphragm preparations. After 90 minutes, the twitch amplitudes were reduced from 100% to 4.5%±2.9% (Figure 1). The mean time required to reduce the twitch amplitudes by 50% (t_1/2_) was 31.9±3.2 minutes. The neuromuscular blockade induced by PrTX-I was not reversed when preparations were washed for 30 minutes with toxin-free physiological solution (data not shown). RA significantly prevented the neuromuscular blockade induced by PrTX-I when both were pre-incubated, but alone the RA did not affect the twitches (Figure 1).

### Muscle-damaging activity

**Figure 2:**
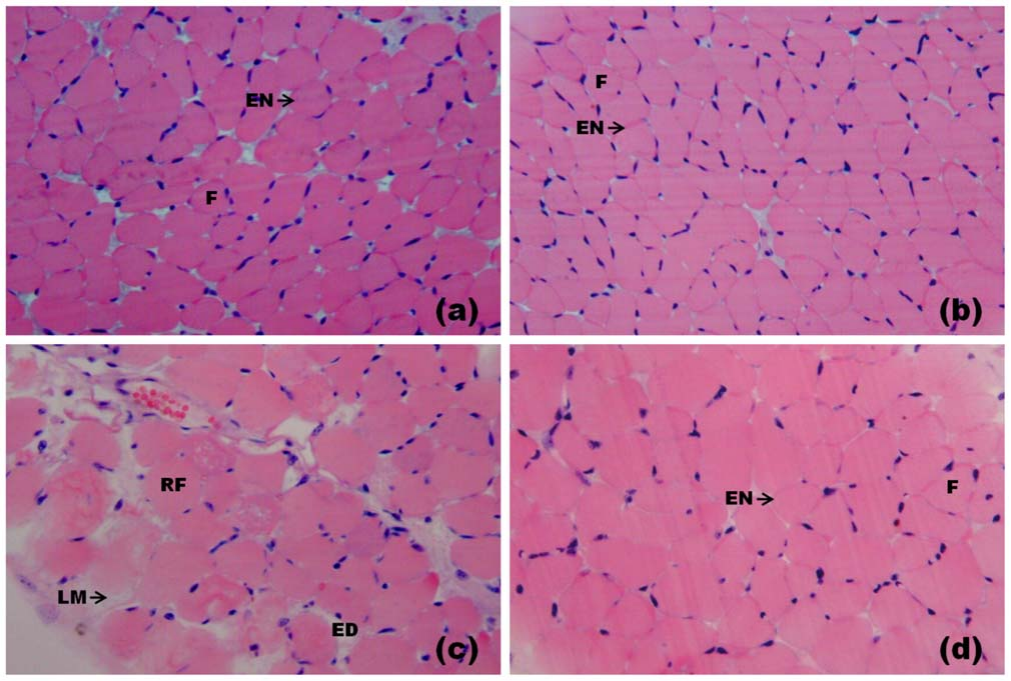
Light micrographs of mouse diaphragm muscles submitted to hematoxilin and eosin staining. Control muscle (a) and muscle exposed to RA (b) show fibers with normal appearance as evidenced by the polygonal aspect of fibers (F) and endomysium (EN). (c) Muscle exposed to PrTX-I: edema (ED), round fibers (RF) some of which present loss of myofibrils (LM). (d) Muscle exposed to PrTX-I pre-incubated with RA: most fibers present a normal appearance.

**Figure 3:**
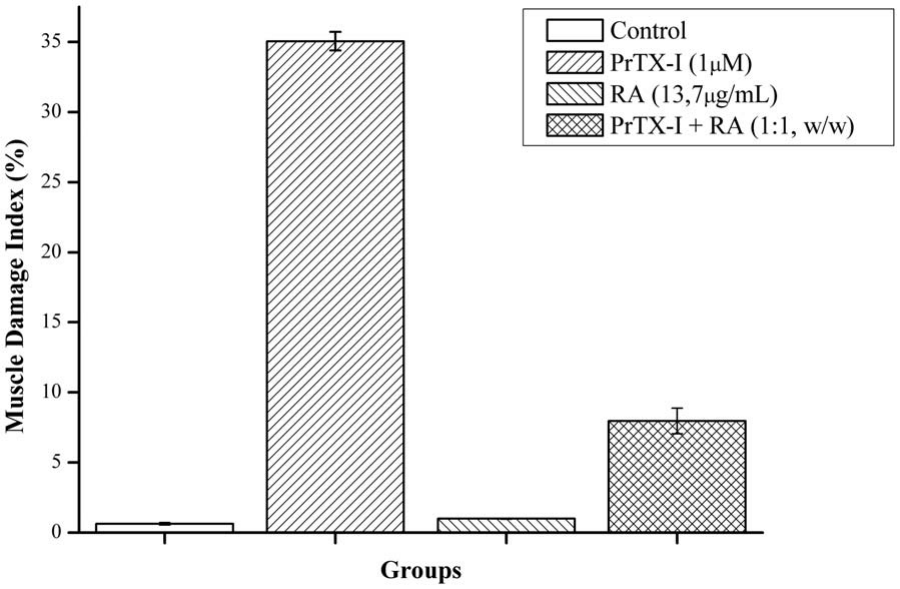
Effect of RA upon the muscle damage index induced by PrTX-I in mouse diaphragm preparations. The ordinate represents the % of damaged fibers relative to normal fibers and the abscissa indicates the experimental groups. The bars are expressed as mean ± S.E.

**Figure 4:**
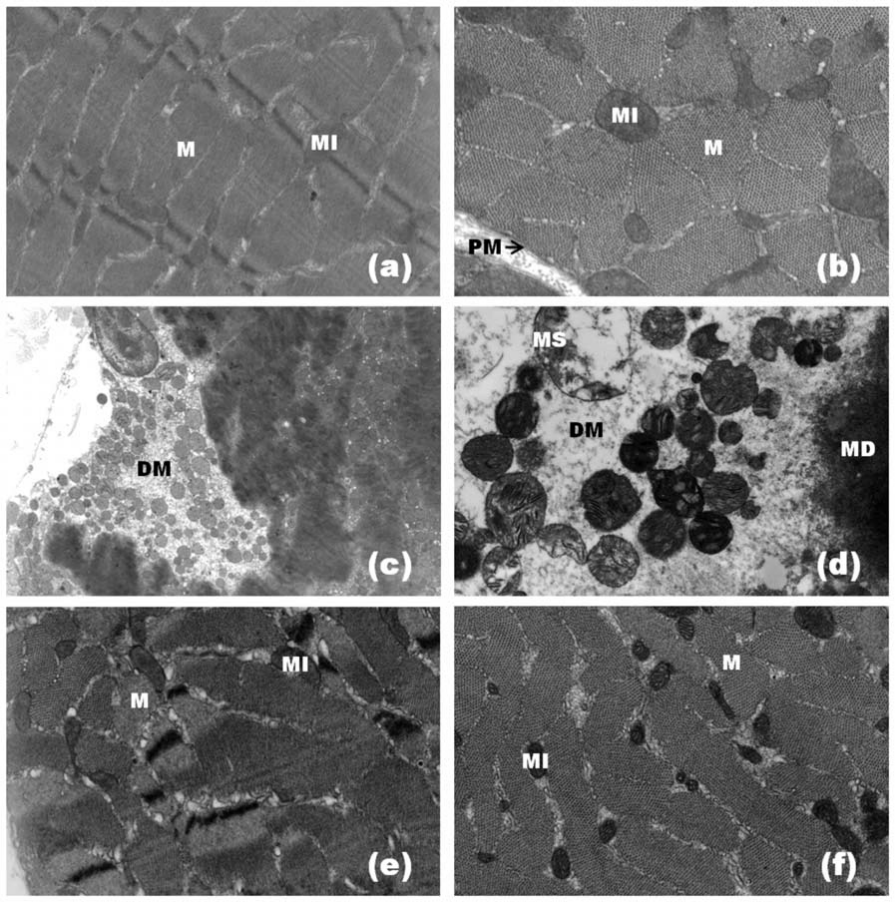
Electron micrographs of mouse diaphragm muscle. Control muscle (a, b) shows normal morphology with plasma membrane (PM), myofibrils (M) and mitochondria (MI). Muscle exposed to PrTX-I (c, d) presents fibers with myofibril disorganization (MD), cytoplasmic areas devoid of myofibrils (DM) and mitochondrial swelling with reduced or ruptured cristae (MS). Muscle exposed to PrTX-I pre-incubated with RA (e, f) shows normal fiber aspect. Note myofibrils (M) and mitochondria (MI) aspects.

Light microscopy showed that a majority of both control (Figure 2a) and RA-exposed (Figure 2b) diaphragm muscle fibers presented normal appearance. Fibers were clearly delimited by a thin layer of connective tissue (the endomysium) and presented a polygonal shape, acidophilic sarcoplasm and peripheral nuclei (Figure 2a, b). Few fibers from the control (0.6±0.1%) and RA-treated muscles (0.9±0.1%) were injured (Figure 3). After 90 minutes of contact with PrTX-I, diaphragm muscle presented fibers with different degrees of damage. The most common features were round fibers and edema in endomysium connective tissue that was characterized by larger spaces between fibers. Many fibers presented cytoplasm areas devoid of myofibrils, some with a central nucleus (Figure 2c). The muscle damage index was 35.1±0.7% (Figure 3). Ultra-structural analysis revealed muscle fibers with loss of myofilaments, leaving sarcoplasmic spaces apparently devoid of myofibrils. Mitochondria showed swelling with reduced or ruptured cristae (Figure 4d). On the other hand, preparations exposed to PrTX-I that had been pre-incubated with RA showed most fibers with normal aspects (Figures 2d, 4e and 4f); the muscle damage index was 7.9±0.9% (Figure 3).

### Crystallographic studies

Crystals of the PrTX-I/RA complex diffracted X-rays to 1.77 Å resolution [61]. Data collection statistics are shown in Table 1. The crystals belong to orthorhombic space group P2_1_2_1_2_1_, with unit-cell parameters a = 49.4 Å, b = 67.0 Å and c = 85.5 Å. The data set is 95.1% complete with an R_merge_ = 6.8%. Calculations based on the protein molecular weight [72] indicate the presence of two molecules in the asymmetric unit [61]. The homodimeric configuration adopted by Lys49-PLA_2_s has already been demonstrated by different techniques [39], [73]–[77] and is assumed to be the biological assembly in this study.

**Table 1:** X-ray data collection and refinement statistics for PrTX-I/RA.

Unit cell (Å)	a = 49.4, b = 67.0, c = 85.5
Space group	P2_1_2_1_2_1_
Resolution (Å)	40-1.77 (1.86-1.77)^a^
Unique reflections	26992 (3895)^a^
Completeness (%)	95.1 (97.5)^a^
R_merge_ b (%)	6.8 (41.2)^a^
Radiation source	Synchrotron (MX2 station, LNLS)
I/σ(I)	16.5 (2.0)^a^
Matthews coefficient V_M_ (Å^3^/Dalton)	2.62
Molecules in asymmetric unit	2
Solvent content (%)	53.12
R_cryst_ c (%)	16.0
R_free_ d (%)	21.7
Mean B-factor (Å^2^)^e^	
Overall	37.2
Protein	22.7
RA molecule	44.2
R.m.s. deviations from ideal values^e^	
bond lengths (Å)	0.022
bond angles (^o^)	2.0
Ramachandran plot^f^	
residues in most favorable/additionally allowed region (%)	89.9/9.1
residues in generously/not allowed regions (%)	1.0/0
Coordinate error (Å)	
SIGMAA (cross-validated SIGMAA)^e^	0.07 (0.08)

a Numbers in parenthesis are for the highest resolution shell.

b R_merge_ = Σ*_hkl_*(Σ*_i_*(|I*_hkl,i_*−<I*_hkl_* >|))/Σ*_hkl,i_* <I*_hkl_*>, where I*_hkl,i_* is the intensity of an individual measurement of the reflection with Miller indices h, k and l, and <I_hkl_> is the mean intensity of that reflection. Calculated for I>−3σ (I).

c R_cryst_ = ∑*_hkl_*(∥Fobs*_hkl_*|-|Fcalc*_hkl_*∥)/|Fobs*_hkl_*|, where |Fobs*_hkl_*| and |Fcalc*_hkl_*| are the observed and calculated structure factor amplitudes.

d R_free_ is equivalent to R_cryst_ but calculated with reflections (5%) omitted from the refinement process.

e Calculated with the program REFMAC [67].

f Calculated with the program PROCHECK [69].

**Figure 5:**
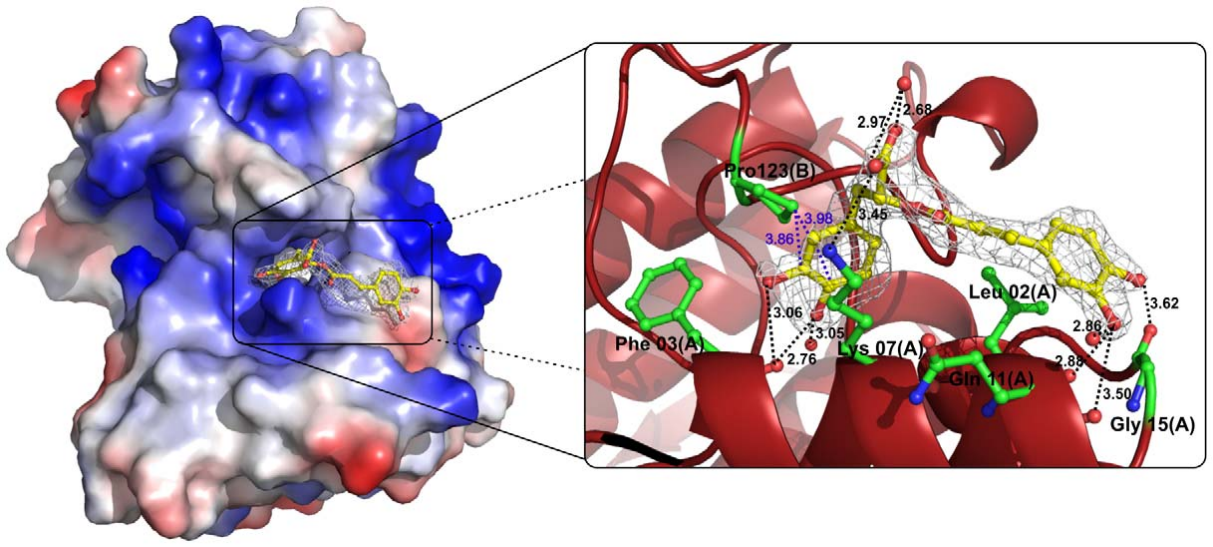
Interactions between RA and PrTX-I atoms in the PrTX-I/RA complex. The surface charge distribution for the PrTX-I/RA crystallographic model and specific interactions between RA with some PrTX-I atoms are shown. Only the interactions with interatomic distances shorten than 3.7 Å are represented between chain A and the RA molecule (black dashes). To represent the interactions between RA and the residue Pro123 of chain B a larger distance cut-off was considered (blue dashes). Residues whose contacts with RA are established through water molecules are not shown. The electron density map for RA molecule was calculated with coefficients 2|F_obs_|-|F_calc_| contoured at 1.0 standard deviation.

**Figure 6:**
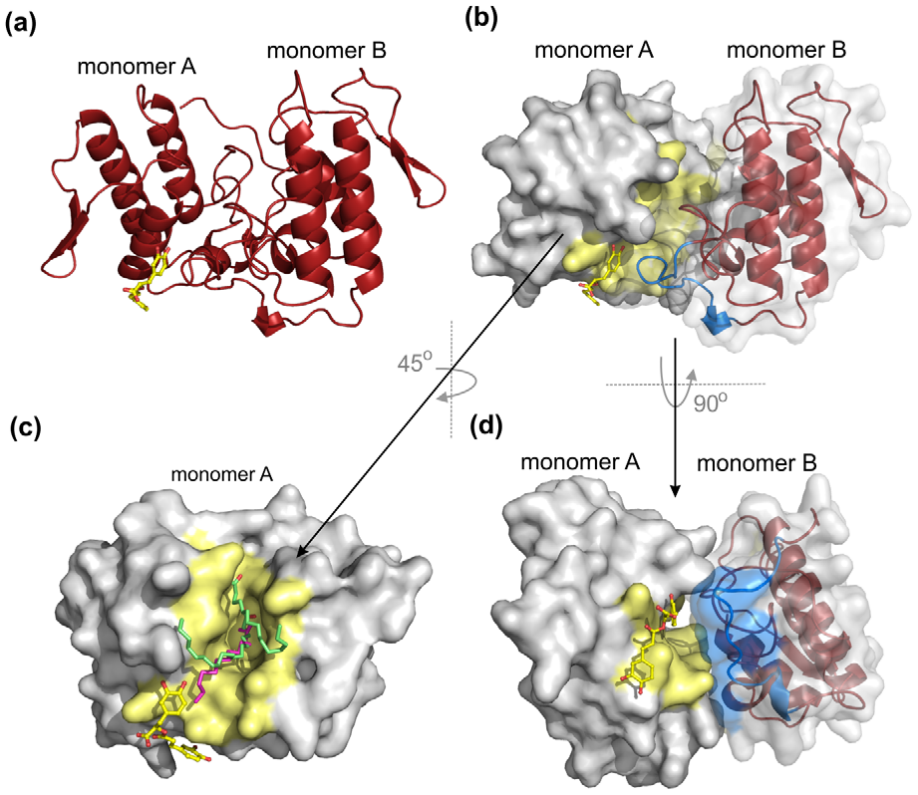
Comparison of the PrtX-I/RA complex with two other Lys49-PLA_2_s complexed to fatty acids. (a) Cartoon representation for the PrTX-I/RA complex. (b) PrTX-I/RA complex displayed in the same orientation shown in (a) shows RA occluding the entrance of the chain A hydrophobic channel. A surface representation is used for protomer A and cartoon representation with a semi-tranparent surface is employed for protomer B. The hydrophobic channel of protomer A is shown in yellow and the C-terminus of protomer B (residues 119–125) is represented in blue. (c) Superposition of PrTX-I/RA complex with PrTX-II/fatty acid (pink) and MjTX-II/stearic acid (light green) complexes (only one protomer of the homodimeric toxins is represented). (d) Surface view of the PrTX-I/RA complex shows that the hydrophobic channel of monomer A is in close contact with the C-terminal region of monomer B. The RA molecule is shown in sticks in all panels.

After molecular replacement and cycles of manual and automated refinement, an electron density that corresponds to an RA molecule was observed at the entrance of the hydrophobic channel monomer A (Figure 5; Figure 6) [61]. Inspection of the |F_obs_|-|F_calc_| electronic density map also showed that one PEG4000 and eight molecules of isopropanol (IOH) also interact with the toxin. RA establishes hydrogen bonds with residues Phe3, Lys7, Leu10, Gln11 and Gly15 of monomer A (Figure 5) and interacts through water molecules with residues Leu2, Arg72 and Trp77 of the same chain. Additionally, interactions between the RA molecule and the residue Pro123 of monomer B are observed (Figure 5). On the other hand, the hydrophobic channel of monomer B is occupied by a PEG4000 molecule. This PEG molecule establishes contacts with Phe3 and Lys7 of monomer B, with the latter being established via a water molecule. Two of the eight isopropanol (IOH) molecules interact with both His48 residues (Nδ1 atoms) of the dimeric structure through the well-known “active-site” water molecule [78]. Asn17:Tyr119 and Tyr119:Tyr119 hydrogen bonds are observed between the protein monomers.

Due to a lack of defined electron density, the side chains of the following residues were modeled as alanine: Lys36, Lys78, Leu116 and Lys129 of chain A, and Lys70, Leu116 and Lys127 of chain B. The side chains of some other residues were modeled as presenting multiple configurations.

**Figure 7:**
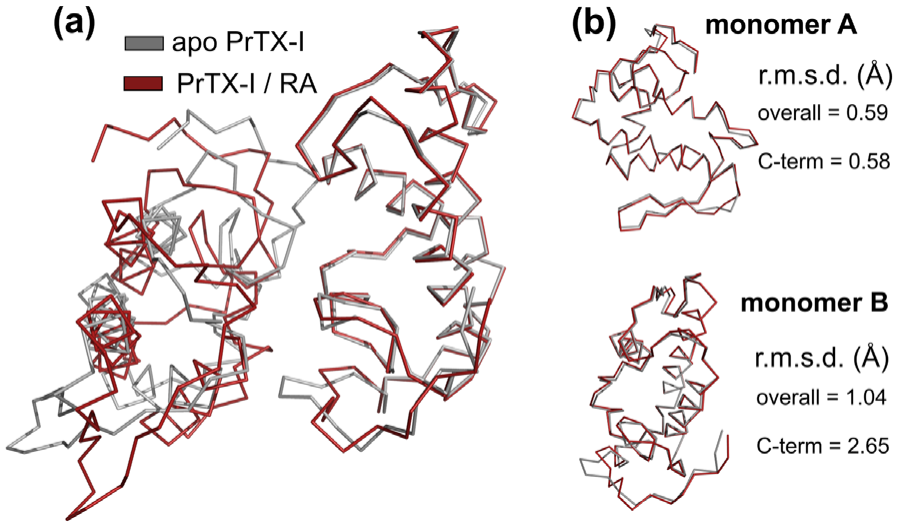
Superposition of the apo and RA-bound forms of PrTX-I. (a) Ribbon representation of both homodimer structures. (b) superposition between corresponding protomers of the structure. The r.m.s.d. values for the whole protomer chain superposition and for the C-terminal region (residues 115–129) are shown. The higher r.m.s.d. for protomer B is due to allosteric changes induced by the ligand in the C-terminal region.

Comparison of the PrTX-I/RA complex with the apo PrTX-I structure [65] (PDB ID 2Q2J) shows that a rearrangement between the monomers is induced by the presence of RA and affects mainly one of the C-termini (overall r.m.s.d. of 1.04 Å and 0.59 Å for protomers A and B, respectively, and 2.65 Å and 0.58 Å for their C-termini, respectively) (Figure 7). This rearrangement can be characterized by the establishment of the Tyr119-Tyr119 interchain hydrogen bond that is a feature common to all structures of Lys49-PLA_2_ complexes solved to date [65], [74], [75], [79].

## Discussion

Myonecrosis is an important consequence of envenomation following *Bothrops* snakebites because it is poorly neutralized by conventional serum therapy and, in severe cases, may lead to amputation and permanent disability [1], [2], [4], [7], [9], [80]. Therefore, there has been a growing interest in the study of venom components involved in the genesis of myonecrosis, their mode of action and the structural basis underlying this biological activity. Several of these investigations have focused on the myotoxic Lys49-PLA_2_ homologues that are widely found in Viperidae snake venoms [15], [81], [82]. One of the experimental strategies used for the study of these myotoxins is based on the evaluation of the functional and structural consequences of their interaction with potential neutralizing agents. Therefore, in this work we investigated the ability of RA to neutralize the muscle-damaging and the neuromuscular-blocking activities of PrTX-I, a Lys49-PLA_2_ from *Bothrops pirajai* snake venom. Additionally, the complex of PrTX-I with RA was crystallized in order to clarify the structural site(s) involved in the toxic effects presented by this Lys49-PLA_2_.

The present data show that RA significantly reduces the blockade of indirectly evoked muscle contractions induced by PrTX-I by up to 90% and inhibits the muscle damage caused by this toxin in isolated phrenic-diaphragm preparations by about 80%. The ability of PrTX-I and several other Lys49-PLA_2_s to block neuromuscular transmission in isolated preparations has been previously described [33]–[38]. However, interpreting this finding has been a challenging task since these myotoxins are devoid of significant neurotoxicity *in vivo* [15]. Nevertheless, a recent review of all the available experimental evidence led to the hypothesis that both the *in vitro* inhibitory neuromuscular effect and the muscle damage promoted by Lys49-PLA_2_s result from their ability to destabilize muscle cell membranes [38]. The first consequence of the muscle membrane destabilization is the collapse of the ionic gradient leading to cell depolarization, probably due to the re-equilibration of Na^+^ and K^+^ ions [83]. In fact, initial contractures and reduction of the resting membrane potential, which indicate membrane cell depolarization, are characteristics of the *in vitro* neuromuscular blockade induced by Lys49-PLA_2_s in frog, chick and mouse preparations [33], [83], [84]. Thus, it has been suggested that the persistent cell depolarization induced by Lys49-PLA_2_s creates areas of membrane inexcitability due to inactivation of voltage-dependent Na^+^-channels, thus impairing the generation of the action potential along muscle fibers [83]. Neuromuscular transmission is highly susceptible to this depolarizing blockade because it depends on the electrical excitability of a restricted membrane area surrounding the endplate region, known as the perijunctional zone [38]. Moreover, the disruption of the muscle fiber membranes induced by Lys49-PLA_2_ homologues also promotes an increase in the concentration of the cytosolic calcium that initiates a complex series of degenerative effects on muscle fibers [38], [85]. The fact that RA considerably reduces both the paralysis and the muscle damage caused by PrTX-I confirms the hypothesis that these effects are closely related.

Rosmarinic acid is a polyphenolic compound found in various plants of the *Boraginaceae* and *Laminaceae* families [86]. Several biological properties have been described for this compound including its ability to neutralize inflammatory, myotoxic and hemorrhagic activities of both crude snake venoms and their isolated toxins [40], [87]. In addition, it has been shown that RA inhibits some enzymes including acetylcholinesterase [88], [89]. However, this anticholinesterase activity occurs within a concentration range that is one to two orders of magnitude higher than that used in our experiments [88], [89] and probably does not explain the ability of RA to neutralize PrTX-I-induced neuromuscular blockade. Additionally, the observation that RA alone does not significantly affect the indirectly evoked contractions supports this idea. Electrophoresis and circular dichroism studies exclude the proteolytic degradation of the toxin as a potential mechanism involved in the inhibition of myotoxic and inflammatory activities of Lys49-PLA_2_s from *Bothrops jararacussu* venom by RA [40]. In view of the abovementioned characteristics, RA is an apt tool for investigating the structural basis underlying the toxic activities of Lys49-PLA_2_s and may even become a potential effective molecule to supplement serum therapy.

The muscle membrane-destabilizing activity exerted by Lys49-PLA_2_s has long been attributed to the presence of basic and aromatic residues situated at specific positions in the C-terminal region of these toxins [15], [55]–[57], [59], [90]–[92] (residues 115–129 according to the numbering system adopted by Renetseder and colleagues [93]). On the other hand, ligands that bind to other sites have also been described in the literature and their ability to diminish the myotoxic effects induced by Lys49-PLA_2_s have been demonstrated [39], [94]–[96].

**Figure 8:**
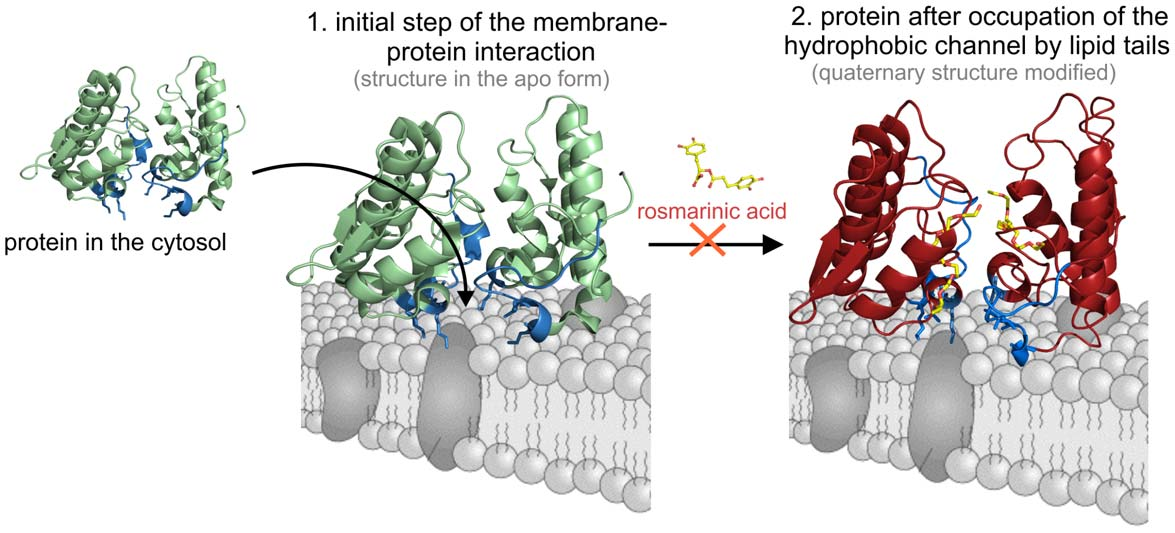
Model for Lys49-PLA_2_ interaction with muscle membrane and the influence of RA. Step 1 represents the initial protein-membrane interactions and step 2 represents the protein after C-termini interactions and insertion of lipid tails in both hydrophobic channels of the toxin [65]. The C-terminal region of the protein (residues 115–129) is colored in blue and residues Lys20, Lys115and Arg118 are highlighted in stick representation. The crystallographic models of BthTX-I (PDB ID 3HZD) and BthTX-I/PEG4000 (PDB ID 3IQ3) are employed for step 1 and 2 representations of the apo and complexed forms of homodimeric Lys49-PLA_2_s, respectively. RA impairs the transition between steps 1 and 2: the inhibitor interacts with the Lys49-PLA_2_ at the entrance of the hydrophobic channels, consequently affecting the ability of lipid tails of membrane phospholipids to be inserted in the toxin hydrophobic channels.

A recent review of all apo and complexed structures of Lys49-PLA_2_s available in the Protein Data Bank (http://www.pdb.org) served as the basis for a prediction of the residues involved in the muscle membrane-destabilizing activity of these proteins (denominated a “myotoxic site”) and a two-step mechanism of action [65] (Figure 8). According to the proposal, the first step of the mechanism is the interaction of Lys20, Lys115 and Arg118 of these proteins with the phospholipid headgroups at the surface of the plasma membrane. Subsequently, a quaternary rearrangement takes place that allows long-chain hydrophobic portions of membrane phospholipids to be inserted into the hydrophobic channel of the toxin [65]. Therefore, the hydrophobic channel was suggested to be involved in one of the steps required for the mechanism of Lys49-PLA_2_s, thereby justifying its conservation among these proteins that do not present any phospholipase activity [65], [75].

The crystal structure of PrTX-I complexed to RA shows that the inhibitor interacts with the toxin at the entrance of its hydropohobic channel (Figure 5; Figure 6), demonstrating for the first time a ligand that interacts with this region of a Lys49-PLA_2_. Considering the mechanism proposed by dos Santos and colleagues [65], this finding suggests that the inhibitory effect of RA is the result of a steric hindrance that blocks the access of substrates to the hydrophobic channel (Figure 6; Figure 8). Supporting this hypothesis, comparison of the PrTX-I/RA complex with structures of two Lys49-PLA_2_s bound to fatty acids (PDB ID 1QLL and 1XXS) [97], [98] suggests that RA impairs the binding of lipid tails to the hydrophobic channel by physically blocking its entrance (Figure 6c). The fact that only one RA molecule was found to interact with the dimeric structure of PrTX-I can be rationalized by the presence of a PEG4000 molecule in the second hydrophobic channel, presumably arising from the high concentration of PEG4000 in the crystallization buffer combined with the high affinity of the channel for hydrophobic molecules.

The way RA interacts with PrTX-I lead us to suggest how the quaternary assembly of the protomers contributes to Lys49-PLA_2_s function. The blockade of one hydrophobic channel of the toxin (protomer A in this structure) by RA molecule occurs when the inhibitor interacts with atoms of both protomers (Figure 5), however conformational changes are observed only in the C-terminus of protomer B (Figure 7).

In conclusion, we have demonstrated that RA interacts with PrTX-I at the entrance of its hydrophobic channel and that the presence of ligand bound outside the well-characterized “myotoxic site” at the C-terminus of Lys49-PLA_2_ affect the toxin's ability to destabilize muscle membranes. Considering the two-step mechanism by which Lys49-PLA_2_s may act [65] it is possible to propose different means to inhibit these myotoxins: i) by inhibition of the “myotoxic site” (e.g. with heparin [99]), ii) by physical inhibition of the hydrophobic channel (e.g. with PEG400 [39]) or by preventing its occupation (e.g. with RA). The combination of these strategies may lead to more successful methods to inhibit bothropic Lys49-PLA_2_s myotoxins within the context of snakebite treatment by the development of inhibitory compounds that may serve as an adjuvant to serum therapy.

### Atomic coordinates

The PrTX-I/RA coordinates have been deposited in the Protein Data Bank with identification code 3QNL.
